# Supplementary material for: An Approach to the Use of Glycol Alkoxysilane–Polysaccharide Hybrids in the Conservation of Historical Building Stones
Source: Molecules. 2021 Feb 10;26(4):938. doi: 10.3390/molecules26040938 (PMC7916683; doi:10.3390/molecules26040938)
Supplement: Supplementary file 1 [file molecules-26-00938-s001.pdf]

Supporting Information

Glycol Alkoxysilanes-Polysaccharides in the Conservation of Historical Building Stones

Miguel Melendez-Zamudio [2], Ileana Bravo-Flores [1], Eulalia Ramírez-Oliva [1], Antonio Guerra-Contreras [1], Gilberto Álvarez-Guzmán [1], Ramón Zárraga-Nuñez [1], Antonio Villegas [1], Merced Martínez-Rosales [1] and Jorge Cervantes [1,*]

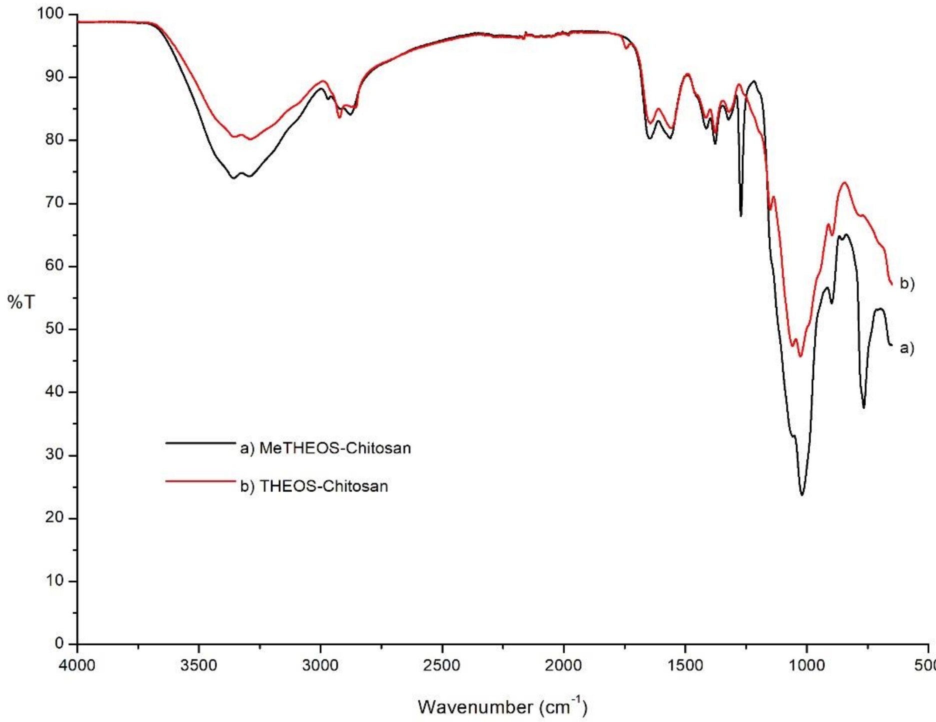

Figure S1. FTIR-ATR for films of (a) MeTHEOS-Chitosan and (b) THEOS-Chitosan

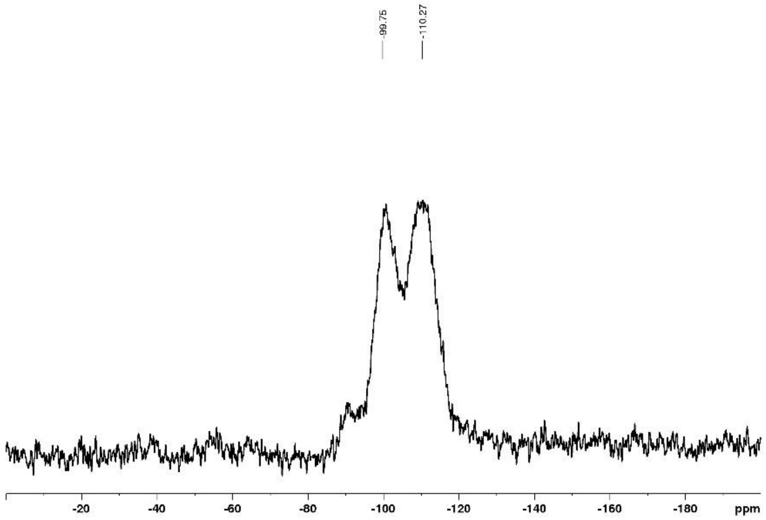

Figure S2. ^{29}Si MAS NMR of THEOS-chitosan film

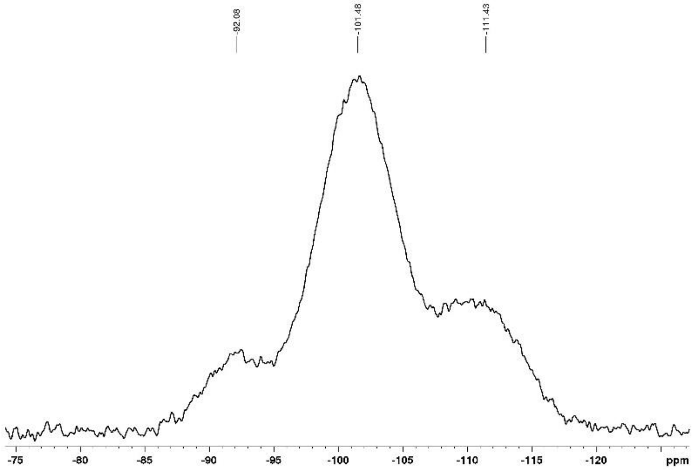

Figure S3. ^{29}Si CPMAS NMR of THEOS-chitosan film

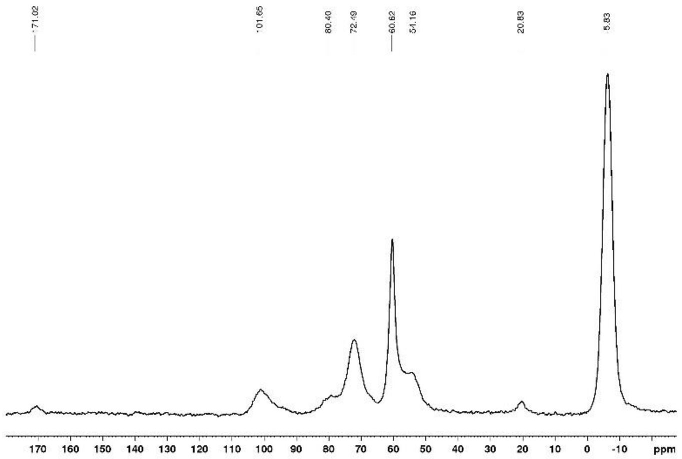

Figure S4. ^{13}C CPMAS NMR of MeTHEOS-chitosan film

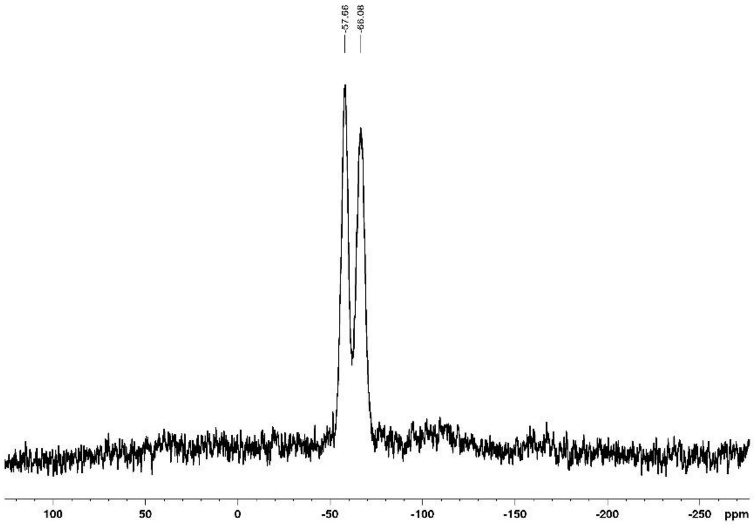

Figure S5. ^{29}Si MAS NMR of MeTHEOS-chitosan film

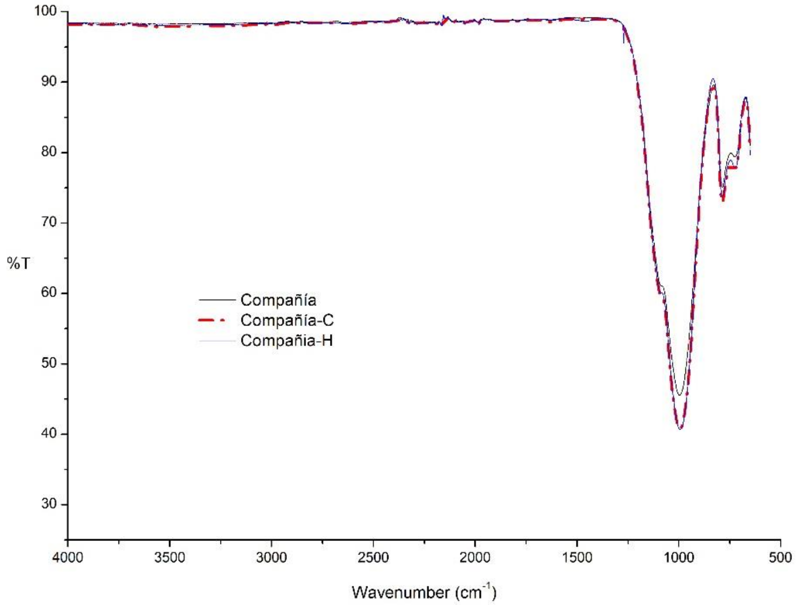

Figure S6. FTIR-ATR spectrum of Compañia sample without treatment, consolidated and hydrofugated.

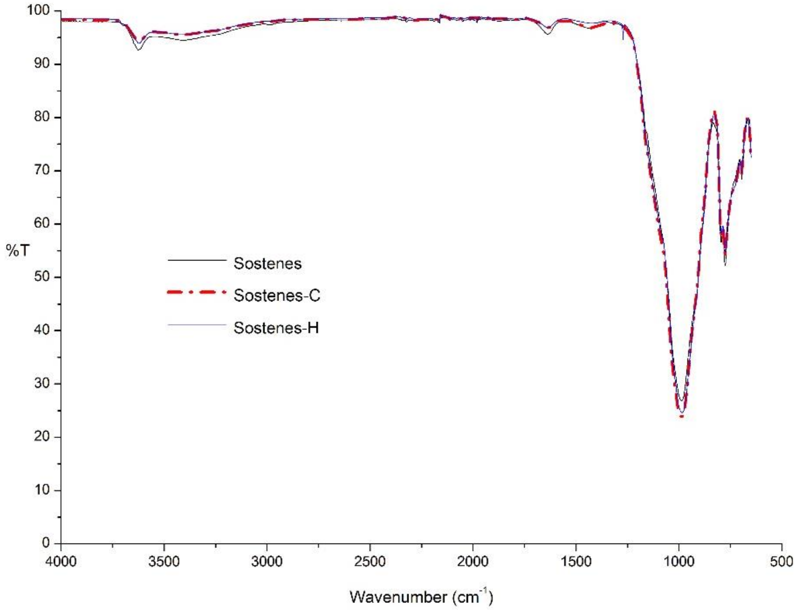

Figure S7. FTIR-ATR spectrum of Sostenes sample without treatment, consolidated and hydrofugated.

Table S.I.1. SEM-EDX elemental composition for MeTHEOS-Chitosan hybrid

Spectrum: MTHQ

Element	Series	unn. C [wt.%]	norm. C [wt.%]	Atom. C [at.%]	Error (3 Sigma) [wt.%]
Oxygen	K-series	41.09	43.96	46.15	15.83
Silicon	K-series	28.08	30.04	17.96	3.56
Carbon	K-series	22.12	23.66	33.08	10.17
Nitrogen	K-series	2.19	2.34	2.81	1.95
	Total:	93.48	100.00	100.00	

Table S2. SEM-EDX elemental composition for THEOS-Chitosan hybrid

Spectrum: THQ

Element	Series	unn. C [wt.%]	norm. C [wt.%]	Atom. C [at.%]	Error (3 Sigma) [wt.%]
Oxygen	K-series	51.60	53.52	55.83	19.86
Silicon	K-series	23.91	24.80	14.74	3.08
Carbon	K-series	17.51	18.16	25.24	8.77
Nitrogen	K-series	3.39	3.52	4.19	2.73
	Total:	96.41	100.00	100.00	

Table S3. SEM-EDX elemental composition for caliche sample without treatment

Element	Series	unn. C [wt.%]	norm. C [wt.%]	Atom. C [at.%]	Error (3 Sigma) [wt.%]
Calcium	K-series	31.69	37.45	18.64	3.37
Oxygen	K-series	41.96	49.58	61.80	16.88
Carbon	K-series	8.57	10.12	16.81	4.18
Silicon	K-series	1.52	1.79	1.27	0.32
Nitrogen	K-series	0.88	1.04	1.49	1.27
	Total:	84.62	100.00	100.00	

Table S4. SEM-EDX elemental composition for consolidated caliche sample

Element	Series	unn. C [wt.%]	norm. C [wt.%]	Atom. C [at.%]	Error (3 Sigma) [wt.%]
Oxygen	K-series	37.02	46.54	49.26	16.76
Calcium	K-series	18.02	22.65	9.57	2.17
Carbon	K-series	19.77	24.86	35.05	9.30
Silicon	K-series	1.40	1.76	1.06	0.34
Nitrogen	K-series	3.33	4.19	5.07	3.61
	Total:	79.55	100.00	100.00	

Table S5. SEM-EDX elemental composition for hydrophobic treated caliche sample

Element	Series	unn. C [wt.%]	norm. C [wt.%]	Atom. C [at.%]	Error (3 Sigma) [wt.%]
Oxygen	K-series	36.35	46.79	45.78	13.54
Carbon	K-series	25.00	32.18	41.94	9.33
Calcium	K-series	10.74	13.83	5.40	1.21
Silicon	K-series	1.63	2.10	1.17	0.30
Nitrogen	K-series	3.97	5.11	5.71	2.54
	Total:	77.68	100.00	100.00	

Table S6. SEM-EDX elemental composition for compañía sample without treatment

Element	Series	unn. C [wt.%]	norm. C [wt.%]	Atom. C [at.%]	Error (3 Sigma) [wt.%]
Oxygen	K-series	23.96	50.78	56.12	10.72
Silicon	K-series	13.58	28.78	18.12	1.84
Carbon	K-series	7.66	16.24	23.91	5.10
Calcium	K-series	1.98	4.20	1.85	0.44
	Total:	47.19	100.00	100.00	

Table S7. SEM-EDX elemental composition for consolidated compañía sample

Element	Series	unn. C [wt.%]	norm. C [wt.%]	Atom. C [at.%]	Error (3 Sigma) [wt.%]
Oxygen	K-series	32.17	55.31	63.81	12.98
Silicon	K-series	21.06	36.21	23.80	2.73
Carbon	K-series	4.02	6.90	10.61	3.16
Calcium	K-series	0.20	0.34	0.16	0.16
Nitrogen	K-series	0.72	1.23	1.62	1.01
	Total:	58.17	100.00	100.00	

Table S8. SEM-EDX elemental composition for hydrophobic treated compañía sample

Element	Series	unn. C [wt.%]	norm. C [wt.%]	Atom. C [at.%]	Error (3 Sigma) [wt.%]
Oxygen	K-series	38.35	55.54	62.86	14.31
Silicon	K-series	23.64	34.23	22.07	3.01
Carbon	K-series	5.91	8.55	12.89	3.56
Nitrogen	K-series	1.16	1.68	2.17	1.13
	Total:	69.06	100.00	100.00	

Table S9. SEM-EDX elemental composition for Sostenes sample without treatment

Element	Series	unn. C [wt.%]	norm. C [wt.%]	Atom. C [at.%]	Error (3 Sigma) [wt.%]
Oxygen	K-series	28.91	55.80	65.61	11.00
Silicon	K-series	18.55	35.79	23.97	2.38
Carbon	K-series	3.06	5.90	9.24	2.17
Calcium	K-series	1.30	2.51	1.18	0.28
	Total:	51.82	100.00	100.00	

Table S10. SEM-EDX elemental composition for consolidated Sostenes sample

Element	Series	unn. C [wt.%]	norm. C [wt.%]	Atom. C [at.%]	Error (3 Sigma) [wt.%]
Oxygen	K-series	56.43	57.62	60.43	20.07
Silicon	K-series	23.10	23.59	14.09	2.93
Carbon	K-series	14.61	14.92	20.85	6.63
Nitrogen	K-series	3.79	3.87	4.63	2.37
	Total:	97.93	100.00	100.00	

Table S11. SEM-EDX elemental composition for hydrophobic treated Sostenes sample

Element	Series	unn. C [wt.%]	norm. C [wt.%]	Atom. C [at.%]	Error (3 Sigma) [wt.%]
Oxygen	K-series	37.88	48.22	46.57	14.90
Carbon	K-series	23.37	29.75	38.27	10.20
Silicon	K-series	12.97	16.51	9.08	1.72
Nitrogen	K-series	4.33	5.52	6.08	3.12
	Total:	78.55	100.00	100.00	
